# On-demand therapies–emergency medication or daily routine?

**DOI:** 10.1007/s00702-026-03288-9

**Published:** 2026-09-24

**Authors:** David Weise, Florin Gandor

**Affiliations:** 1 Department of Neurology, Asklepios Fachklinikum Stadtroda, Bahnhofstr. 1a, 07646 Stadtroda, Germany; 2 Department of Neurology, University of Leipzig, Leipzig, Germany; 3 Department of Neurology, Macquarie University Health, Sydney, Australia; 4 Faculty of Medicine, Health and Human Sciences, Macquarie University, Sydney, Australia; 5 Department of Neurology, St. Vincent’s Hospital Sydney, Sydney, Australia; 6 Movement Disorders Hospital, Kliniken Beelitz GmbH, Beelitz-Heilstätten, Beelitz, Germany; 7 Department of Neurology, Otto-von-Guericke University, Magdeburg, Germany

**Keywords:** Parkinson’s disease, Motor fluctuations, Non-motor fluctuations, Wearing-off, Early morning OFF, On-demand therapy, Apomorphine, Inhaled levodopa

## Abstract

Fluctuations represent a major therapeutic challenge in Parkinson’s disease (PD) and become increasingly prevalent with disease progression. While oral and device-aided therapies (DAT) improve symptom control, patients continue to experience disabling OFF episodes, in particular early morning OFFs, delayed ON, and no-ON phenomena. Increasing evidence suggests that both motor and non-motor fluctuations are more common than previously recognized and contribute substantially to impaired quality of life. Fluctuations affect up to 50% of patients within five years of disease onset to eventually affect almost all patients with longer duration. Beside altered central pharmacodynamics, peripheral mechanisms such as dysphagia, gastroparesis, altered intestinal absorption, and microbiome-mediated levodopa degradation, contribute to fluctuations. Although oral adjunct medication and DAT reduce daily OFF-time, clinically meaningful OFF periods often persist. This is where on-demand therapies provide rapid symptom relief and address the limitations of conventional oral medication and DAT. Hence, more regular, even daily use of on-demand therapies may improve quality of life in patients with PD. However, the potential impact of on-demand therapies on non-motor OFF periods needs further investigation. Side effects, handling and economic considerations should be taken into account. This expert perspective concludes that given the high prevalence and clinical relevance of fluctuations, on-demand therapies may be considered as an integral component of standard PD management rather than solely as a rescue option in rare emergency situations. Implementation may be particularly beneficial for patients experiencing early-morning-OFFs, delayed and no-ONs but also predictable and unpredictable OFFs.

## Introduction

The introduction of levodopa transformed the management of Parkinson’s disease (PD) and remains the cornerstone of its symptomatic treatment (Höglinger and Trenkwalder 2024; Riederer et al. 2025). However, despite its unparalleled efficacy, the progression of the disease is frequently accompanied by the development of response fluctuations. Motor fluctuations (MF) represent the hallmark of disease progression in PD. Their prevalence steadily increases over time, eventually affecting most patients receiving dopaminergic therapy. Furthermore, fluctuations represent a particularly important therapeutic challenge because they significantly impair functional independence, reduce quality of life, and increase healthcare utilization (Chapuis et al. 2005; Politis et al. 2010; Stocchi et al. 2014).

The increasing recognition of MF and non-motor fluctuations (NMF), together with advances in non-oral therapy options, have prompted reconsideration of current treatment paradigms. In particular, on-demand therapies have emerged as valuable tools for the management of acute OFF episodes and may warrant earlier and more regular use than traditionally recommended.

This expert perspective gives an overview of the epidemiology, detection, clinical significance, and treatment of fluctuations in PD, focusing on the role of on-demand therapies and their integration into routine clinical practice.

### Motor fluctuations (MF)

Historically, MF were considered a relatively late manifestation in the course of the disease. However, contemporary evidence suggests that MF develop substantially earlier than previously appreciated (Schrag and Quinn 2000; Stacy 2010). In a longitudinal cohort study, the cumulative incidence of MF reached 54% within five years and 100% within ten years after disease diagnosis (Kim et al. 2020). Earlier studies reported MF in approximately 20% of patients after only nine months of levodopa treatment and in as many as 75% after only three years depending on the levodopa dose (Fahn et al. 2004; Olanow et al. 2013). More recently, data from the LEAP study demonstrated that approximately 36% of patients developed MF within five years regardless of whether levodopa treatment was initiated early or late, suggesting that disease progression rather than levodopa exposure alone drives its development (Verschuur et al. 2019; Frequin et al. 2024). This is further supported by a study comparing patient populations with immediate and delayed access to levodopa therapy, concluding that MF and dyskinesias are not associated with the duration of levodopa therapy, but with longer disease duration. In addition, higher levodopa daily dose was associated with a higher risk of MF (Cilia et al. 2014). A recent meta-analysis including seven studies comparing early vs. delayed levodopa treatment in PD concluded that delaying treatment with levodopa does not prevent levodopa-related motor complications in PD (Ramanzini et al. 2024). Hence, young-onset PD patients are affected by response fluctuations earlier in their life (Kostic et al. 1991). Thus, MF have to be considered as somewhat mandatory in the course of PD, even when considering differences in age at onset, disease duration, levodopa daily dose, and survival time. The clinical significance of MF extends beyond impaired mobility. OFF periods interfere with activities of daily living, increasing dependence on caregivers, and are associated with significantly reduced quality of life (Chapuis et al. 2005; Politis et al. 2010; Stocchi et al. 2014), even if the type, duration, and severity of OFF symptoms may vary during the day and between patients (Pahwa et al. 2023).

### Non-motor fluctuations (NMF)

In addition to motor manifestations, fluctuations frequently involve non-motor symptoms (Storch et al. 2013; Martinez-Fernandez et al. 2016). Approximately 40% of patients experience NMF after eight to ten years of disease duration with fatigue being the most prevalent symptom (Rodriguez-Blazquez et al. 2020). Further symptoms include anxiety, depression, pain, autonomic dysfunction, and cognitive impairment and may occur simultaneously with MF (Rodriguez-Blazquez et al. 2020).

However, the analysis of timing and kinetics of NMF in detail revealed a lack of temporal connection with MF. Close temporal correlation was only shown for mood and cognitive symptoms (Ossig et al. 2017). This temporal disconnection may explain the difference in response thresholds of dopaminergic treatment for motor and non-motor symptoms and therefore becomes highly relevant regarding on-demand treatment of NMF. Those non-motor symptoms that are linked to motor OFF episodes might therefore respond to on-demand therapies that is administered for motor off symptoms. It can only be speculated if different thresholds responses would implicate higher dosing for non-motor OFF episodes. Importantly, numerous studies have shown NMF to be equally or even more disabling than motor symptoms, both for PD patients and their caregivers (Lubomski et al. 2021; Ledda et al. 2025). The complexity of NMF contributes to its frequent under-recognition and underestimation of their impact on overall disease burden and highlights the need for systematic assessment.

### Detection of fluctuations

Recognition of fluctuations remains challenging in routine clinical practice. In order to capture and quantify motor complications such as fluctuations and dyskinesia the MDS-UPDRS part IV is the gold standard for objectifying motor complications in both daily routine and clinical studies (Goetz et al. 2008). In addition, structured screening instruments such as the Wearing-Off Questionnaire (WOQ-19) identify MF and NMF more effectively than conventional physician questioning (Stocchi et al. 2014). Even the shorter version (WOQ-9) may be a useful and an easy to perform screening tool for detecting wearing-OFF in PD (Stacy et al. 2008). Hauser developed the PD Home Diary, a patient-reported tool developed to track and quantify motor fluctuations and dyskinesia in patients with PD (Hauser et al. 2004). It has recently been found that the self-assessed detection of OFF periods compared to clinical assessment correlated well, whereas dyskinesia did not (Löhle et al. 2022). However, due to the differing temporal patterns of NMF and MF, conventional screening tools may not be sensitive enough to detect NMF, which leads to the conclusion that NMF should be assessed separately (Ossig et al. 2016). The introduction of wearables for monitoring MF has further changed the understanding of their prevalence. In a large cohort of 3,288 patients monitored using the Personal KinetiGraph^®^ (PKG^®^), PKG-data suggested that morning OFF periods were present in 85% of patients, and 64% to have a delayed or incomplete response to the morning levodopa dose (Isaacson et al. 2022a). These findings suggest that fluctuations may be substantially more frequent than previously recognized by conventional clinical assessment even if an algorithmically detected morning bradykinesia or a suboptimal treatment response may not be necessarily equivalent to a clinician- and patient-confirmed OFF episode.

### Types and causes of fluctuations

The pathophysiology of motor fluctuations is multifactorial. While progressive degeneration of nigrostriatal dopaminergic neurons reduces the buffering capacity for exogenous levodopa and narrows the so-called therapeutic window, peripheral pharmacokinetic factors have emerged as additional important contributors to fluctuating treatment responses (LeWitt 2015; Politi et al. 2018). Wearing-OFF is characterized by a gradual loss of symptom control at the end of the levodopa dosing cycle, often accompanied by a delay in achieving benefit from the next levodopa intake (delayed ON; Olanow et al. 2021). Early morning-OFF (EMO) and wearing-OFF represent the first, most common and clinically relevant manifestations of response fluctuations, especially in the mid-stage of PD. In a multicenter study on 320 patients with a mean disease duration of seven years, approximately 60% reported EMO symptoms. Notably, EMO encompasses both motor symptoms as well as non-motor manifestations such as urinary urgency, pain and anxiety (Rizos et al. 2014). Morning-OFFs are often severe and frequently accompanied by a delayed-ON, making them particularly suitable for an on-demand therapy in the morning (Isaacson et al. 2022a).

The gastrointestinal dysfunction represents another important contributor to response fluctuations. Dysphagia may impair medication intake and contribute to no-ON episodes (Suttrup and Warnecke 2016; Labeit et al. 2022). While it was believed for a long time that dysphagia is a late-stage symptom (Poewe et al. 2017), recent studies revealed that dysphagia affects PD patients rather early (Patel et al. 2020; Pflug et al. 2018). Furthermore, in the course of the disease, gastroparesis can contribute to a delayed gastric emptying and consequently postpones delivery of levodopa to the small intestine, where its absorption predominantly occurs. This contributes to post-prandial OFF, delayed ON, incomplete ON, and no-ON phenomena (Pfeiffer et al. 2020; Damiens et al. 2026). Additional factors include competition between amino acids and levodopa for absorption as well as microbiome-related levodopa degradation. Small intestinal bacterial overgrowth (SIBO) has been associated with reduced levodopa bioavailability and may contribute to delayed or incomplete medication response (Beckers et al. 2022). Collectively, these mechanisms emphasize that fluctuations are frequently driven by impaired drug transport and absorption rather than insufficient dosing alone. Therefore, addressing gastrointestinal dysfunction including such as dysphagia or constipation can improve response fluctuation and should be considered before adjusting oral therapy.

### Potential of oral add-on medication and device-aided therapies

Current treatment strategies for motor fluctuations initially focus on optimization of oral dopaminergic therapy. Since MF and NMF frequently correlate, this treatment strategy is also applied for NMF. Adjunctive treatment with catechol-O-methyltransferase inhibitors, monoamine oxidase-B inhibitors, dopamine agonists, and adenosine A2A receptor antagonists (only approved in USA and Japan) can reduce OFF time by approximately 0.5–2.5 h per day, also depending on patient selection and disease severity, baseline treatment, definitions and measurement of OFF time, duration of follow-up, control groups and placebo effects and concomitant medication adjustments (Stowe et al. 2011; Antonini et al. 2018; Armstrong and Okun 2020; de Bie et al. 2025). However, patients included in these studies had around 3–6 h OFF time at baseline (Antonini et al. 2018; de Bie et al. 2025). Hence, although these improvements are clinically meaningful, patients enrolled in pivotal studies typically continued to experience several hours of daily OFF time despite optimized therapy. For patients with more severe fluctuations, DAT such as deep brain stimulation, levodopa-carbidopa intestinal gel infusion, continuous subcutaneous apomorphine infusion, and continuous subcutaneous foslevodopa/foscarbidopa infusion offer additional therapeutic benefit. Studies have reported reductions in daily OFF time between 2.5 and 4 h resulting in better motor performance and quality of life in study populations with 6–7 h OFF time at baseline (Olanow et al. 2014; Katzenschlager et al. 2018; Soileau et al. 2022; Antonini et al. 2022; Fabbri et al. 2023). Again, even if these DAT show relevant improvement in OFF time and more stable levodopa plasma concentrations (Rosebraugh et al. 2021), patients may continue to experience clinically significant residual OFF periods.

### On-demand therapy: a paradigm shift?

Traditionally, on-demand therapies have been viewed primarily as rescue medications for unpredictable (motor) OFF episodes. However, given the quite high residual OFF time and prevalence of OFF periods, respectively, despite adjunct medication and DAT, this concept is being increasingly challenged (Isaacson et al. 2022b; Pahwa et al. 2023). A more contemporary treatment paradigm considers on-demand therapies as a parallel strategy rather than a late-stage rescue option. Such an approach may be particularly relevant for patients experiencing EMO, predictable and unpredictable OFF periods (such as post-prandial OFF), delayed ON, or no-ON episodes. Furthermore, on-demand therapies may be useful when patients anticipate the need for an improved motor function during specific activities with a high dopaminergic demand such as exercising or participating in social events (Regensburger et al. 2026). Earlier integration as well as more regular use of on-demand therapies could therefore provide a more comprehensive symptom control. Independent from gastrointestinal dysfunction and food intake on-demand therapies may improve quality of life even if central mechanisms are not addressed (Isaacson et al. 2022b; Pahwa et al. 2023). Non-motor OFF episodes, at least symptoms that often accompany dopaminergic motor OFF episodes (such as pain and anxiety; Rizos et al. 2014; Ossig et al. 2017) may also benefit from on demand therapies. However, the evidence for treating NMF with these interventions remains limited. Furthermore, it has be mentioned that current on-demand therapies are not approved for (pure) non-motor OFF episodes.

### Types of on-demand therapies

Rapid-release (dispersible/soluble) oral levodopa (Madopar© LT/rapid, Isicom©) is still among the most frequently prescribed rescue medication (in Europe and Asia) even if its evidence from randomized clinical studies is limited (de Bie et al. 2025; Höglinger et al. 2025). This robust clinical experience is supported by a recent study where dispersible levodopa as the first morning dose shortened the time to ON compared to regular levodopa (24 vs. 35 min; Chang et al. 2025). However, soluble levodopa does not bypass the gastrointestinal tract and still depends on enteral absorption. Currently, three non-oral on-demand therapies are available: subcutaneous apomorphine, sublingual apomorphine, and inhaled levodopa (see Table 1).

Subcutaneous apomorphine remains the fastest and most potent on-demand treatment with availability in most countries (Olanow et al. 2021; Castillo-Torres et al. 2023). Studies have demonstrated rapid motor improvement, with responses typically occurring within 5–25 min and robust improvements in motor scores up to 60 min. Up to 95% of patients respond to apomorphine, and the therapy is particularly effective for EMO (Dewey et al. 2001; Pahwa et al. 2007; Pfeiffer et al. 2007; Olanow et al. 2021). Subcutaneous injection of apomorphine proves faster symptom relief compared to soluble levodopa and therefore has been considered the “drug of choice for this treatment modality” (Merello et al. 1997). However, treatment initiation requires dose titration, and therapeutic use involves pen and skin preparation. Furthermore, this dopamine agonist can be associated with group-specific adverse effects such as orthostatic hypotension, impulse control disorders, excessive daytime sleepiness, hallucinations, and peripheral edema, respectively. Furthermore, adverse effects more specific to apomorphine comprise of nausea and vomiting and injection-site reactions. (Dewey et al. 2001; Olanow et al. 2021; Pahwa et al. 2023). For the titration process premedication with domperidone is recommended, additionally requiring mandatory ECG controls. Treatment with concomitant 5-HT3 antagonists including antiemetics such as ondansetron is contraindicated. This may be the reason why despite its very quick and reliable efficacy its use has not enjoyed widespread practice in the PD community (Poewe and Wenning 2000; Olanow et al. 2021).

Thus, the sublingual form of apomorphine provides a non-invasive alternative, available in Germany and select European markets, but not in Australia, US or Canada. Studies have demonstrated rapid onset of action within 30 min and sustained efficacy for up to 90 min (Olanow et al. 2020; Kassubek et al. 2024), which is a faster response than conventional levodopa (Hui et al. 2020; Isaacson et al. 2023). The clinical efficacy and tolerability did not differ between sublingual and subcutaneous application of apomorphine, but patients expressed greater satisfaction with the sublingual route as reported in an open-label, randomized, crossover study (Stocchi et al. 2023). The absence of injections and the lack of a strict requirement for in-clinic titration may improve convenience (Kassubek et al. 2023). Furthermore, the most effective and best tolerated dose is usually found within in the first 3 dosings (Hui et al. 2020). Antiemetic premedication is not routinely required. However, study withdrawal due to systemic side effects such as nausea and local side effects such as lip swelling, mouth ulceration and stomatitis was quite high (Kassubek et al. 2024). Hence, oral mucosal adverse events and nausea remain important limitations and require active monitoring (Olanow et al. 2020, 2021). Therefore, the initiation of the first application (usually 10 mg) should be performed in the controlled environment of a specialist clinic under the supervision of an appropriately trained healthcare professional.

Inhaled Levodopa formulation (Inbrija^®^) is approved in the US and select European countries for the intermittent treatment of OFF episodes in PD patients treated with oral levodopa. The approved dosage is two capsules of 42 mg each provision of respirable dose of 33 mg of levodopa each and taken PRN up to five times per day. It is administered via a breath-actuated inhaler and rapidly absorbed through the pulmonary system, bypassing the gastrointestinal tract (LeWitt et al. 2019; Paik 2020; Jost et al. 2023). Patients must remain on a stable levodopa/dopa-decarboxylase inhibitor regimen (as the capsule does not include a dopa-decarboxylase inhibitor). Inhaled levodopa as a single dose of 50 mg administered during an OFF episode resulted more often in clinically relevant levodopa plasma concentrations (> 400 ng/ml) compared to oral levodopa in PD patients (77 vs. 27%; Lipp et al. 2016). Another study confirmed that inhaled levodopa powder was more rapidly absorbed, resulting in higher plasma concentrations within the first 45 min than oral levodopa. Furthermore, it resulted in lower inter-patient response variability than the oral alternative (Safirstein et al. 2020). Clinically meaningful motor improvement is usually achieved within 10 min and lasts up to 60 min, with its peak after around 30 min. Advantages include the absence of injections and dose titration requirements. However, correct inhaler technique is essential, cough is a common adverse effect, and dosing flexibility is limited (LeWitt et al. 2019; Grosset et al. 2020). Therefore, treatment is not recommended in patients with asthma, COPD or another chronic underlying pulmonary disease. Furthermore, the adequate inhalation technique can be challenging for patients in an OFF state (Jost et al. 2023). Hence, it is mandatory that patients are adequately trained.

Clinically meaningful and rapid relief during particular episodes may indeed not necessarily result in a large reduction in total daily OFF time as seen in the study on inhaled L-Dopa (LeWitt et al. 2019). However, the strength of on-demand therapies lies in its short latency to ON, a high probability of achieving a full ON and very good motor improvement with a long duration (of at least 60 min; Pahwa et al. 2007; LeWitt et al. 2019; Olanow et al. 2020; Olanow et al. 2021).

### Challenges of on-demand therapies

The subcutaneous, mucosal and pulmonary delivery of these new on-demand therapies provides a more predictable and faster treatment effect, independent of gastrointestinal dysfunction or food intake. Importantly, there currently exist no head-to-head comparisons between available on-demand therapies. Consequently, treatment selection must be individualized and guided by efficacy, tolerability, patient preference, speed of onset, and practical considerations (LeWitt and Martinez-Nunez 2023). Furthermore, the effectiveness of on-demand therapies depends on the patient’s ability to recognize an evolving OFF episode early and administer the therapy promptly and correctly (Olanow et al. 2021; LeWitt and Martinez-Nunez 2023; Regensburger et al. 2026). This is usually easier to embed into daily routine than in an emergency situation, e.g. a sudden OFF episode. Many patients are not accustomed to non-oral on-demand therapies in clinical routine, probably due to the need of dose titration (apomorphine subcutaneous pen and sublingual strip), specific side effects (apomorphine subcutaneous pen and sublingual strip, levodopa inhalation powder) and difficulties in handling (levodopa inhaler, apomorphine subcutaneous pen and sublingual strip). Hence, the introduction and use of non-oral on-demand therapies require consideration and careful education (see Table 2).

**Fig. 1:**
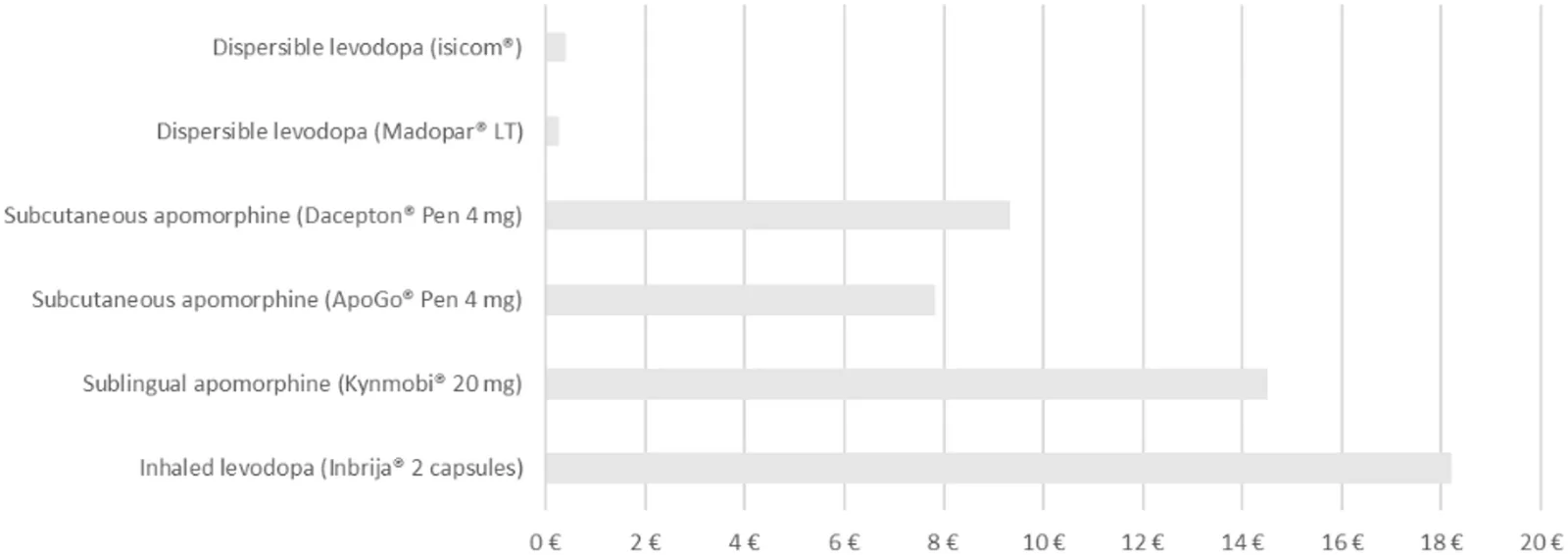
Price of a (common) single dose of on-demand therapies in Germany (costing based on current recommended retail prices for largest pack size in Germany as of June 2026; single doses are not necessarily therapeutically equivalent)

Economic considerations are increasingly relevant when selecting amongst available on-demand therapies. Cost analyses suggest substantial differences between treatment options, with costs varying several-fold. While soluble levodopa formulations remain inexpensive, newer proprietary on-demand therapies, especially sublingual apomorphine and levodopa inhalation powder are associated with considerably higher treatment costs (see Fig. 1). These economic considerations should be balanced against potential reductions in disability, healthcare utilization, and caregiver burden.

## Conclusion

MF and NMF are highly prevalent throughout the course of PD and occur more frequently than previously recognized. Advances in structured assessment and wearable monitoring technologies have revealed the high prevalence and substantial burden of OFF periods, particularly EMO, delayed ON, and no-ON phenomena. Current evidence indicates that gastrointestinal dysfunction plays an important role in the pathogenesis of fluctuations by impairing levodopa transport and absorption. Although oral adjunctive therapies and DAT reduce OFF time, many patients continue to experience clinically relevant fluctuations for several hours per day. Most on-demand therapies bypass the critical gastrointestinal barrier and thereby provide rapid symptomatic relief. Their role should therefore extend beyond that of emergency rescue medications. Instead, on-demand therapies may be incorporated into routine clinical management and considered earlier, particularly in patients with EMO, delayed ON, predictable OFF periods, and no-ON episodes. Future studies should determine the optimal positioning of on-demand therapies within modern treatment algorithms of MF and NMF and clarify their long-term impact on patient outcomes.

**Table 1:** Approved on-demand therapies for the treatment of OFF episodes in patients with Parkinson’s disease

	Route of administration	Usual/typical and maximum dose/frequency	Approximate onset and duration	Need for supervised titration	Common adverse effects	Major contraindications and precautions	Handling requirements	Regional availability
Dispersible/soluble levodopa (Madopar^®^ LT/rapid, Isicom^®^)	Oral	50–100 mg, no max. dose/frequency	Onset 30–45 min, duration 60–90 min	No	Nausea, dizziness, orthostatic hypotension (in general less common)		Water for dissolve required	Most countries
Apomorphine hydrochloride injection (Apokyn^®^, APO-go^®^, Dacepton^®^)	Subcutaneous	4–6 mg, max. single/daily dose 20/30 mg	“Onset 10–15 min, duration 45–60 min”	Yes (in- and outpatient clinic)	Nausea, vomiting, orthostatic hypotension, somnolence; injection-site reactions	5-HT3 antagonists	Premedication recommended (domperidone)	Most countries
Apomorphine sublingual film (Kynmobi^®^)	Sublingual	15–20 mg, max. single dose 30 mg, max. 5x/day	Onset 15–30 min, duration 60–90 min	Yes (initial dose under supervision; possible titration at home)	Nausea, vomiting, oropharyngeal reaction	5-HT3 antagonists		Select European countries
Levodopa inhalation powder (Inbrija^®^)	Inhaled	2 capsules, max. 5x/day	“Onset 15–30 min, duration 45–60 min”	No	Cough, nausea, sputum discoloration	Pulmonary diseases	Training recommended, decarboxylase-inhibitor in regular medication required	US and select European countries

**Table 2:** Therapy recommendation for specific clinical symptoms

Clinical symptom	On-demand therapy			
	Dispersible/soluble levodopa (Madopar^®^ LT/rapid, Isicom^®^)	Apomorphine hydrochloride injection (Apokyn^®^, APO-go^®^, Dacepton^®^)	Apomorphine sublingual film (Kynmobi^®^)	Levodopa inhalation powder (Inbrija^®^)
Predictable wearing-OFF	+*	+*	+*	+*
Delayed or absent response to an oral dose (no ON)	-	++	++	++
Early-morning OFF	++	++	++	+
Dysphagia or suspected gastroparesis	-	++	++	++
Unpredictable OFF	+	++	++	++
Impaired manual dexterity°	+	-	+	-
Cognitive impairment	++	+	+	+
Pulmonary disease	+	+	+	--
Hallucinations	++	-	+	++
Impulse control disorders	++	+	+	++
Orthostatic hypotension	++	-	-	++

-- Contraindication, - not recommended, + possible option, ++ recommended; * check optimization of regular medication; ° no caregiver available

## Data Availability

No datasets were generated or analysed during the current study.
